# Localized Delivery of Pilocarpine to Hypofunctional Salivary Glands through Electrospun Nanofiber Mats: An Ex Vivo and In Vivo Study

**DOI:** 10.3390/ijms20030541

**Published:** 2019-01-28

**Authors:** Sujatha Muthumariappan, Wei Cheng Ng, Christabella Adine, Kiaw Kiaw Ng, Pooya Davoodi, Chi-Hwa Wang, Joao N. Ferreira

**Affiliations:** 1 Department of Oral and Maxillofacial Surgery, Faculty of Dentistry, National University of Singapore, Singapore 119085, Singapore; email2sujatha227@gmail.com (S.M.); christabella.adine@u.nus.edu (C.A.); kiawkiaw.ng@gmail.com (K.K.N.); 2 Department of Chemical and Biomolecular Engineering, National University of Singapore, Singapore 117585, Singapore; weicheng.ng@gmail.com (W.C.N.); davoodi.pooya@u.nus.edu (P.D.); chewch@nus.edu.sg (C.-H.W.); 3 Faculty of Dentistry, Chulalongkorn University, Bangkok 10330, Thailand; 4 National Institute of Dental and Craniofacial Research, National Institutes of Health, Bethesda, MD 20892-4370, USA

**Keywords:** electrospinning, nanomaterials, nanofibers, drug delivery, pilocarpine, salivary glands, hypofunction, dry mouth, xerostomia

## Abstract

Dry mouth or xerostomia is a frequent medical condition among the polymedicated elderly population. Systemic pilocarpine is included in the first line of pharmacological therapies for xerostomia. However, the efficacy of existing pilocarpine formulations is limited due to its adverse side effects and multiple daily dosages. To overcome these drawbacks, a localized formulation of pilocarpine targeting the salivary glands (SG) was developed in the current study. The proposed formulation consisted of pilocarpine-loaded Poly(lactic-*co*-glycolic acid) (PLGA)/poly(ethylene glycol) (PEG) nanofiber mats via an electrospinning technique. The nanofiber mats were fully characterized for their size, mesh porosity, drug encapsulation efficiency, and in vitro drug release. Mat biocompatibility and efficacy was evaluated in the SG organ ex vivo, and the expression of proliferation and pro-apoptotic markers at the cellular level was determined. In vivo short-term studies were performed to evaluate the saliva secretion after acute SG treatment with pilocarpine-loaded nanofiber mats, and after systemic pilocarpine for comparison purposes. The outcomes demonstrated that the pilocarpine-loaded mats were uniformly distributed (diameter: 384 ± 124 nm) in a highly porous mesh, and possessed a high encapsulation efficiency (~81%). Drug release studies showed an initial pilocarpine release of 26% (4.5 h), followed by a gradual increase (~46%) over 15 d. Pilocarpine-loaded nanofiber mats supported SG growth with negligible cytotoxicity and normal cellular proliferation and homeostasis. Salivary secretion was significantly increased 4.5 h after intradermal SG treatment with drug-loaded nanofibers in vivo. Overall, this study highlights the strengths of PLGA/PEG nanofiber mats for the localized daily delivery of pilocarpine and reveals its potential for future clinical translation in patients with xerostomia.

## 1. Introduction

Dry mouth syndrome or xerostomia is a common global health problem that arises most commonly within an ageing population [1,2,3]. At the age of 70, 16% of men and 25% of women report xerostomia symptoms [2]. Xerostomia is mostly triggered by diuretic medication regimens, radiotherapy for head and neck cancers, auto-immune disorders, and uncontrolled diabetes, among other diseases [4,5]. Currently, muscarinic agonists like pilocarpine are the first line of pharmacological therapy for xerostomia [4]. Pilocarpine is only available in oral formulations, which upon ingestion produce significant systemic adverse effects, leading to low tolerability and reducing patient adherence to the drug [6,7]. Furthermore, pilocarpine tablets are contra-indicated in many medical conditions affecting the elderly, including cardiovascular disease, chronic obstructive pulmonary disease, and glaucoma [4,7]. Only half of the patients respond well to pilocarpine tablets, probably due to its short duration of action (3–5 h), requiring multiple daily administrations [8]. To this end, a localized pilocarpine formulation, specifically targeting the SG and bypassing systemic adsorption, is mandatory to make the saliva secretion more long-lasting. A recent double-blind randomized clinical trial showed that 0.1% pilocarpine mouthwash was not more effective than 0.9% saline after four weeks of treatment [9]. However, when pilocarpine dosing is increased to 1–2% in studies using mouthwash, there is a significant increase in salivation and high tolerability due to the absence of adverse side effects [10,11]. Several studies have tested the efficacy of topical pilocarpine administration via mouthwash or oral rinses, but they show contradicting outcomes [8,12], perhaps because pilocarpine mouthwashes only target the minor SG present in the oral mucosa, which are glands that in normal conditions do not significantly contribute to the whole saliva output (<1%) [13]. Major SG located underneath the facial skin should be targeted instead, potentially via an intradermal or transdermal route [14]. To meet this need, a new localized and SG-targeted pilocarpine drug delivery system is needed.

Poly(lactic-*co*-glycolic acid) (PLGA) and poly(ethylene glycol) (PEG) are advantageous materials for use as drug carriers due to their favorable biodegradability and biocompatibility, which ensure safe therapies [15,16]. Various carriers in the form of particles and fiber meshes fabricated in our previous works using electrohydrodynamic atomization (EHDA) techniques have been proven to work effectively for sustained and controlled drug release with hydrophilic drugs like pilocarpine [17,18]. In EHDA, the solution is subjected to high voltage, and the charged droplet disintegrates into fine jets or aerosols in a Taylor cone-like fashion, which upon immediate evaporation of the solvent leads to the formation of the desired solid carriers [19,20]. EHDA can be operated in the electrospraying or electrospinning mode to obtain different products (particles or fibers) of different sizes (microns, sub-microns, etc.) and morphologies, depending on the operating parameters (flow rates, voltage, etc.) and properties of material used (concentration, electrical conductivity, surface tension, viscosity, etc.) [21,22]. For instance, highly concentrated and viscous liquid favors the electrospinning regime to form fibers, as the liquid easily solidifies at the onset of jetting owing to a sufficiently strong elastic network that stabilizes the jet against breakup [22].

In this study, we present a new approach for the localized and controlled delivery of pilocarpine and successfully evaluated it ex vivo and in vivo. The proposed formulation was composed of homogenous pilocarpine-loaded PLGA/PEG nanofiber mats using electrohydrodynamic atomization techniques in electrospinning mode. Next, we aimed to (1) characterize the diameter distribution, porosity and in vitro drug release of this localized nanofiber mat formulation, to (2) evaluate its biocompatibility in ex vivo SG organs, and to (3) compare the in vivo saliva secretion rates of this localized formulation against systemic pilocarpine.

## 2. Results

### 2.1. Fabrication of Electrospun Nanofiber Mats

**Figure 1:**
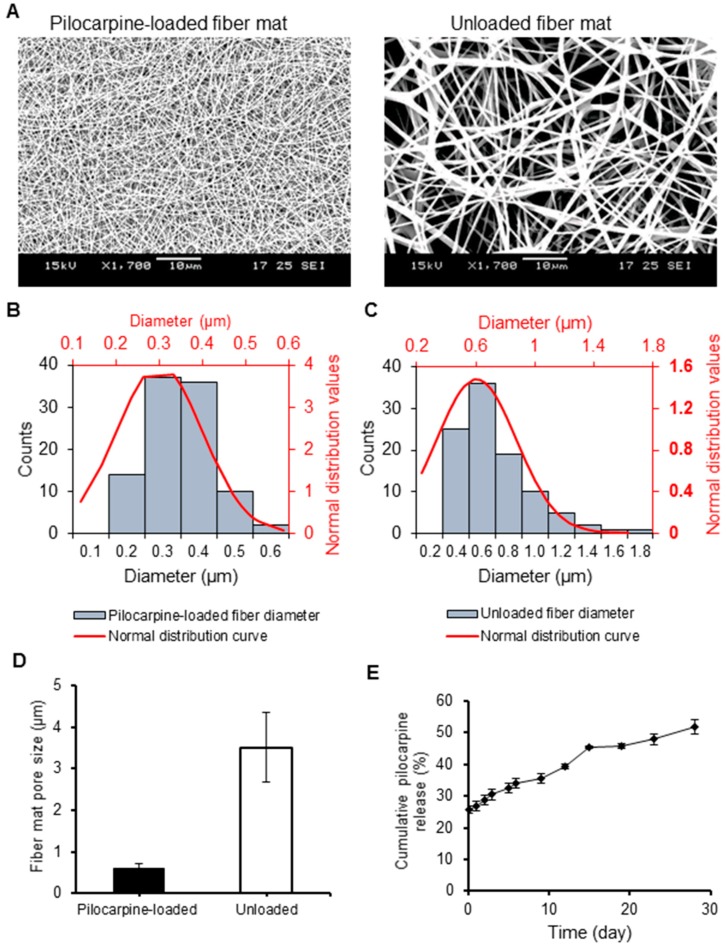
Structural and morphological comparison of pilocarpine-loaded and unloaded PLGA nanofiber mats, and in vitro pilocarpine release profile from pilocarpine-loaded mats. (**A**) Scanning electron microscopy (SEM) images at 1700× magnification. (**B**–**C**) Diameter distribution of the loaded (left) and unloaded PLGA fiber mats (right). (**D**) Mean porosity distribution of PLGA fiber mats. Error bars represent SD from *n* = 10. (**E**) Cumulative pilocarpine drug release from loaded PLGA nanofibers in the short and long term supported a steady pilocarpine release in vitro. The observed drug release profile was about 26% in the first 4.5 h, and increased steadily to 36% after 9 d, to 45% after 15 d, and to 52% after 28 d. This supports a steady pilocarpine release in vitro. Error bars represent SD from *n* = 3.

After optimization, it was found that using acetone as the solvent gave the most homogeneous and stable polymer solution/emulsion, compared to other organic solvents tested. At PLGA concentration of <30% (*w*/*v*), the fibers produced were intertwined with unwanted particles. Changes in the collection height and voltage did not significantly change this outcome, though increasing the PLGA concentration from 10% to 25% (*w*/*v*) did augment the ratio of fibers to particles. Only at 30% (*w*/*v*) PLGA concentration were uniform nanofibers with no more particles consistently observed (Figure 1A), and this was accomplished with electrospinning operating parameters of 0.3 mL/h flow rate, 11.0 kV voltage, and 10 cm height of collection. Our finding was consistent with the literature in that, in general, a lower polymer concentration favors the formation of particles while a higher polymer concentration favors fiber fabrication [23].

### 2.2. Physical and Chemical Characterization of Nanofibers

#### 2.2.1. Scanning Electron Microscopy (SEM)

SEM images of the fabricated PLGA fiber mats (pilocarpine-loaded and unloaded) can be observed in Figure 1A. The average diameter and porosity of the fibers were determined for the loaded and unloaded formulations (Figure 1B,C, respectively). Well-distributed nano-scale fibers were observed for the pilocarpine-loaded fiber mat (diameter 384 ± 124 nm), and submicron fibers for the unloaded fiber formulation (diameter 936 ± 258 nm). The average porosity (pore size) of the pilocarpine-loaded fiber mat was significantly smaller as compared to the unloaded (611 ± 107 nm versus 3510 ± 838 nm) (Figure 1D). The porosity and diameter of the loaded nano-scale fiber mat was uniform as per its limited standard deviation. 

#### 2.2.2. Drug Loading Capacity, Encapsulation Efficiency, and Degradation

Next, the drug loading capacity of the loaded PLGA nanofiber mats as well as the encapsulation efficiency was calculated based on spectrophotometry results and standard theoretical formulas mentioned above. The drug loading capacity of the nanofibers was determined to be 0.84 ± 0.09% and the encapsulation efficiency was 81.1 ± 8.4%. For degradation studies of pilocarpine solution in different diluents (MilliQ water and 1× PBS), the optical densities of pilocarpine across a period from 4.5 h to 2 d did not change and were comparable for both diluents (results not shown), which indicates that no drug degradation occurred. 

### 2.3. Short- and Long-Term In Vitro Pilocarpine Release

To determine the pilocarpine release from the PLGA nanofiber mats through time, we measured the pilocarpine released into a shaking PBS buffer from 4.5 h up to 28 d. In Figure 1E, the release curve showed an initial burst release of one-fourth of the encapsulated pilocarpine (25.7%) after 4.5 h, followed by a rather gradual and steady release of pilocarpine over time. After 15 d, almost 50% of encapsulated pilocarpine was released. Then, a slight plateau was noted from day 15 to day 19. After day 19, there was only a 6% increase up to day 28.

### 2.4. Ex Vivo Studies in Hypofunctional Salivary Glands

#### 2.4.1. Cytotoxicity and ATP Activity of Pilocarpine

**Figure 2:**
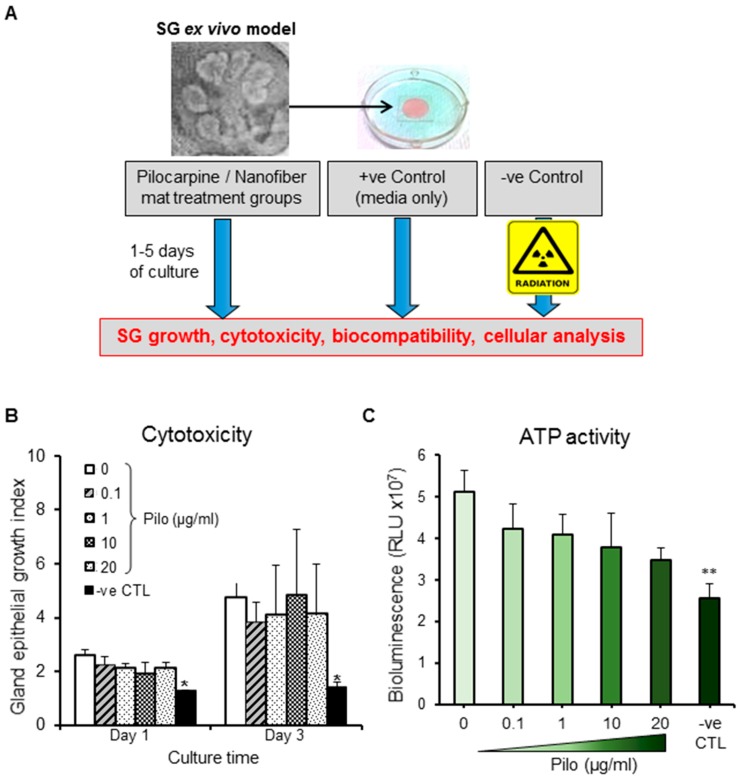
Biological effects in an ex vivo SG model. (**A**) Ex vivo SG model experimental set up used for testing the cytotoxicity of pilocarpine and biocompatibility of different nanofiber mats. (**B**) Glands were treated with therapeutic concentrations of pilocarpine (Pilo) ranging from 0.1 to 20 µg/mL. All gland growth index values at day 1 and 3 were normalized to time 0 (baseline). (**C**) ATP activity (a readout for gland viability) three days after glands were treated with therapeutic concentrations of pilocarpine (0.1–20 µg/mL). Error bars represent SD from *n* = 4. * *p* ˂ 0.05 and ** *p* ˂ 0.01 when compared to positive control without pilocarpine (0 μg/mL). Negative control (-ve CTL) represent glands damaged by gamma radiation.

In order to test the cytotoxicity of pilocarpine in the SG organ, we cultured salivary glands ex vivo with media supplemented with this drug (Figure 2A) at a range of clinically relevant therapeutic concentrations, according to previous reports [24,25]. All pilocarpine concentrations, ranging from 0.1 to 20 µg/mL, supported the glandular growth through 48 h (Figure 2B and Figure S1), as well as the cellular metabolism as per stable ATP activity (Figure 2C). This indicated that no major organ cytotoxicity is induced by the pilocarpine solution within this clinical range. Therefore, the loaded nanofibers were fabricated to maximize the amount of pilocarpine up to 20 µg/mL.

#### 2.4.2. Biocompatibility of Nanofiber Mats (Loaded and Unloaded)

**Figure 3:**
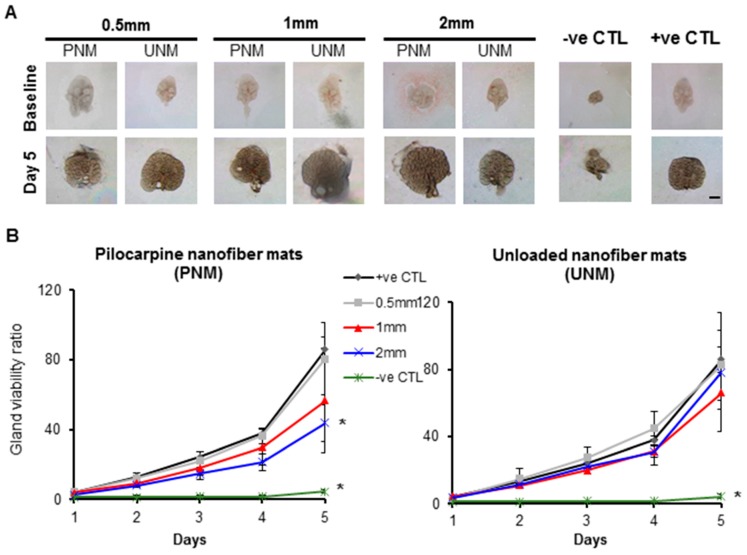
Biocompatibility of pilocarpine-loaded and unloaded nanofiber mats (PNM and UNM, respectively) in the ex vivo SG culture model. (**A**) Bright field images of the growing SG glands at 3.2× magnification. Scale bar: 400 µm. (**B**) SG epithelial viability (readout for organ biocompatibility) was supported by unloaded nanofibers and by 0.5 mm and 1 mm loaded nanofibers. Y axis is a ratio of epithelial bud number at a specific culture time relative to baseline. Error bars represent SD from *n* = 4–12. * *p* ˂ 0.05, when compared to positive control without pilocarpine (+ve CTL) at every culture day; ns: not significant when compared to “+ve CTL”. Negative control (-ve CTL) represent glands damaged by gamma radiation. PNM: Pilocarpine nanofiber mats. UNM: unloaded nanofiber mats.

To test the biocompatibility of the nanofibers, three different nanofiber discs were produced with different diameters and with a maximized concentration of pilocarpine (20 µg/mL), as shown in Figure S1. Then, ex vivo SG were cultured with media supplemented with pilocarpine-loaded and unloaded discs, and gland viability (a readout of epithelial growth) was quantified in the short and long term (Figure 3A,B). There was a steady increase in SG viability and growth in all treatment groups throughout the 5 d of culture. However, the 2 mm pilocarpine-loaded nanofiber mats produced a statistically significant lower gland viability across all culture days as compared to the gland with only media and no treatment provided (+ve CTL). 

#### 2.4.3. Biological Effects of Pilocarpine-Loaded Nanofiber Mats in SG Cellular Compartments

**Figure 4:**
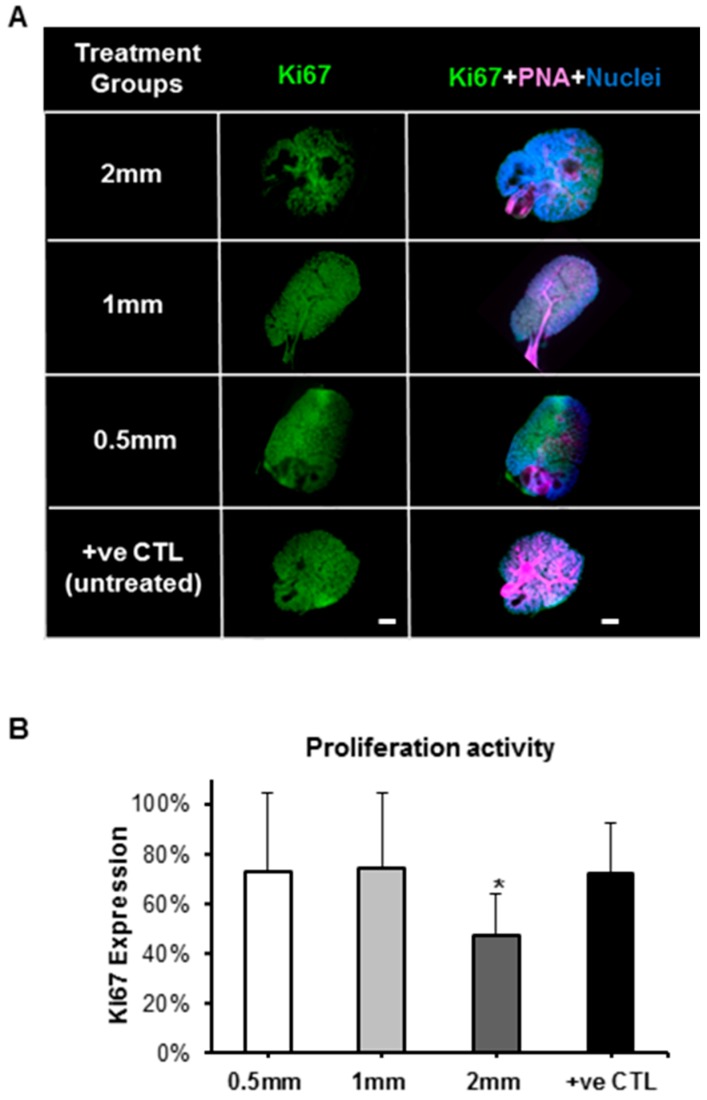
Expression of proliferation protein marker (Ki67) in SG after treatment with pilocarpine nanofiber mats after five culture days in the ex vivo SG model. (**A**) Fluorescence imaging after whole gland immunofluorescence staining showing expression of Ki67 in green (pro-mitotic marker), PNA in pink (peanut agglutinin staining the gland epithelial acini and ductal branched network) and nuclei in blue. (**B**) Expression of proliferation/pro-mitotic activity by quantification of Ki67 fluorescence after normalizing with nuclear counts. Error bars represent SD from *n* = 4. * *p* ˂ 0.05 when compared to positive control. Positive control (+ve CTL) was not treated with nanofiber mats.

**Figure 5:**
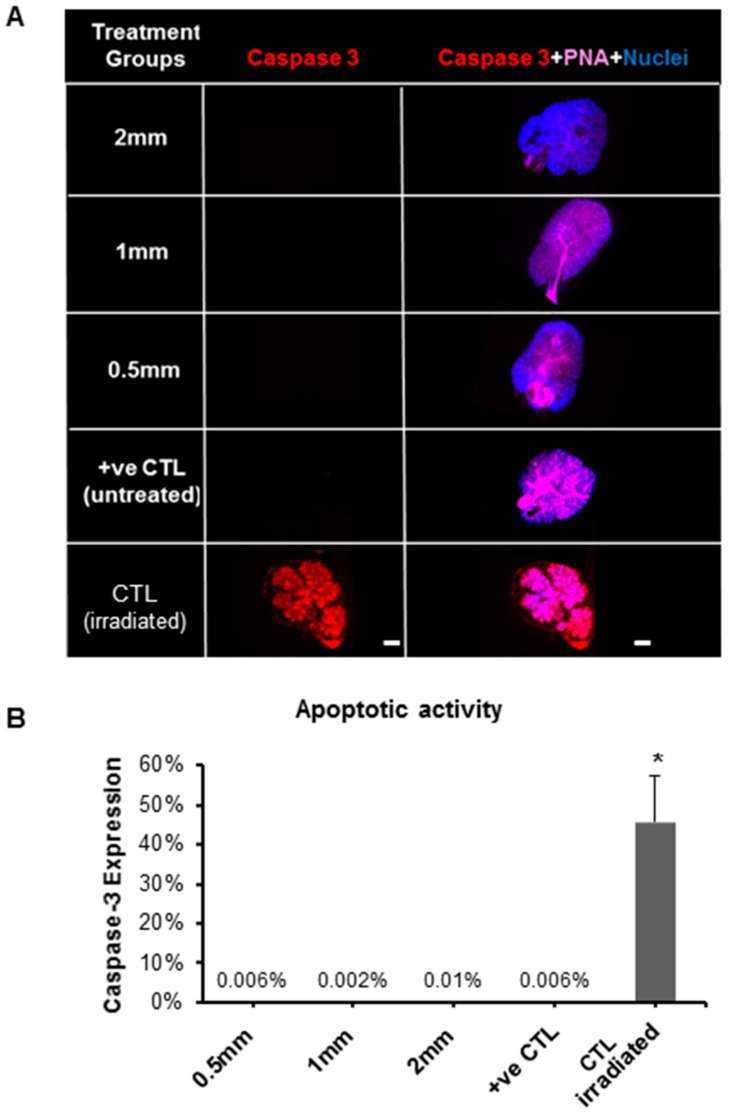
Expression of apoptotic protein marker (Caspase-3) in SG after treatment with pilocarpine nanofiber mats after five culture days in the ex vivo SG model. (**A**) Fluorescence imaging after whole gland immunofluorescence staining showing expression of Caspase-3 in red (apoptotic marker), PNA in pink (staining the gland epithelial acini and ductal branched network) and nuclei in blue. (**B**) Apoptotic activity by quantification of Caspase-3 fluorescence after normalizing with nuclear counts. Error bars represent SD from *n* = 4. * *p* ˂ 0.05 when compared to positive control. Positive control (+ve CTL) was not treated with nanofiber mat and only had growth media. CTL (irradiated): gamma radiation treatment was used as a control for Caspase-3 staining since it induces apoptotic damage to the gland.

Next, ex vivo salivary glands treated with pilocarpine-loaded nanofiber mats for 5 d were analyzed for the expression of cellular proliferation/pro-mitotic (Ki67) and pro-apoptotic (cleaved Caspase 3) markers, as seen in Figure 4 and Figure 5, respectively. The Ki67 expression confirmed that the epithelial proliferation significantly decreased, in both the acinar branches and ductal networks, with 2 mm loaded discs (Figure 4 and Figure 5). These cellular-based findings confirmed the outcomes from the previous organ toxicity experiments. As for cleaved Caspase 3, the expression of this pro-apoptotic marker was low (≤0.01%), and no statistical differences were observed between the loaded discs. This suggests that salivary gland pro-apoptosis is grossly absent in the presence of the pilocarpine-loaded mats ranging from 0.5 mm to 2 mm, regardless of the pilocarpine amount in such mats.

### 2.5. In Vivo Saliva Secretion after Intradermal Treatment with Pilocarpine-Loaded Nanofiber Mats versus Systemic Pilocarpine

**Figure 6:**
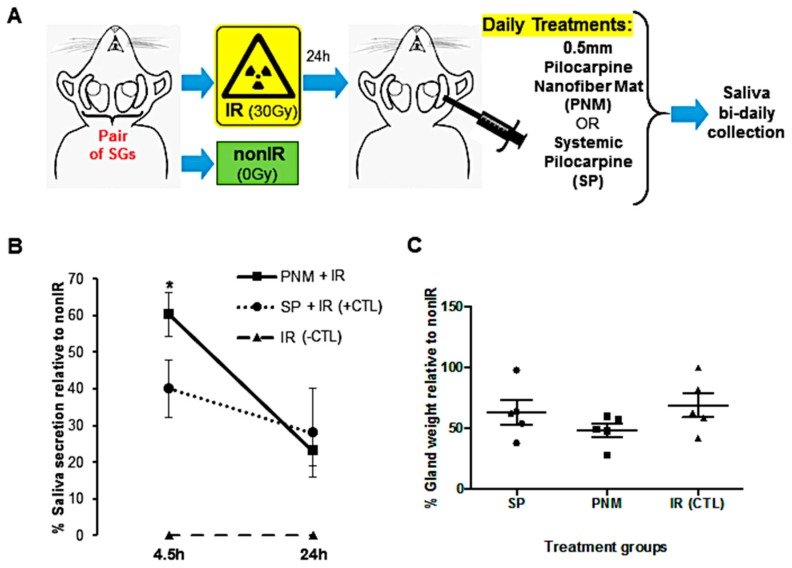
Treatment outcomes during daily intradermal applications of 0.5 mm pilocarpine nanofiber mats versus systemic pilocarpine in an acute in vivo SG model of SG hypofunction. (**A**) Schematic drawing of the in vivo SG hypofunction model to induce acute dry mouth for the daily proposed intradermal treatment with pilocarpine nanofiber mat and conventional systemic administration. (**B**) Saliva secretion rate during the first 24 h after daily intradermal application of 0.5 mm-diameter pilocarpine nanofiber mats (PNM) when compared to systemic pilocarpine (SP). (**C**) Salivary gland weight remained unchanged after intradermal application of 0.5 mm pilocarpine nanofiber mats. Error bars represent SEM from *n* = 4–5. * *p* ˂ 0.05 when compared to irradiated group with systemic pilocarpine only (SP, which represents the positive CTL). IR: irradiated (negative CTL). nonIR: non-irradiated control group. SGs: salivary glands.

Lastly, in vivo hypofunctional SG in an acute model (Figure 6A) were treated with intradermal applications of pilocarpine-loaded nanofiber mats (0.5 mm-diameter mats were selected based on high biocompatibility rates ex vivo) and compared with systemic pilocarpine (oral pilocarpine formulation could be used as per IACUC and mouse model limitations), and the salivary flow rate was determined to test our novel intradermal application formulation on a daily basis for 24 h (Figure 6B). After 4.5 h, SG with intradermal pilocarpine-loaded mats significantly increased the saliva secretion when compared to the systemic pilocarpine (Figure 6B). After 24 h, no difference was noticed between these two treatment formulations (Figure 6B). Furthermore, the whole gland weight was comparable between the two formulations indicating no gross changes in SG composition and cellular content (Figure 6C). Also, no histological differences were found between treatment groups.

## 3. Discussion

This study developed a novel localized pilocarpine intradermal drug delivery system supporting the treatment of xerostomia at a greater extent than systemic pilocarpine. Our electrohydrodynamic atomization technique supported the production of a homogenous porous mats with a well-distributed network of electrospun PLGA/PEG nanofibers. Previous ophthalmic pilocarpine formulations used similar ingredients but a double emulsion fabrication method [24]. This latter method facilitated the fabrication of loaded nanoparticles, which possessed a pilocarpine encapsulation efficiency of only 57% as compared to 81.1% with our nanofibers. For double emulsion fabrication method, the chaotic liquid flow in the stirring region often results in spatially non-uniform stress and poor monodispersity, which is known to lead to low encapsulation efficiency [26]. In contrast, such stirring is not required in EHDA, and the fabrication is a one-step process as opposed to two-stage, hence minimizing the loss of drugs. This is also one key feature of the EHDA technique. Unfortunately, the oral/ophthalmic pilocarpine formulations could not be used in our mouse SG hypofunctional model as per local IACUC policies and unpredictable survival outcomes.

The nanofiber mats fabricated herein had a homogenous morphology, consistent diameter and porosity, alike other reports using electrospun fibers (Figure 1A–D) [27,28,29]. Other methodologies like the dropping method in a study by Kao and colleagues [25] revealed the difficulties in controlling the size of pilocarpine-loaded nanoparticles composed of chitosan and carbopol. In contrast, the nano-scale diameter of our fabricated pilocarpine-loaded mats was achievable with lesser difficulties probably due to salts present in the pilocarpine hydrochloride solution used. These salts might have enhanced the overall solution conductivity leading to nano-scale fiber formation when subjected to high voltage, as compared to the sub-micron scale of unloaded fiber mats [30].

As for the drug release profile (Figure 1E), a biphasic pilocarpine release in our nanofiber mats delivery system could be observed. The first phase consisted of an initial burst release of more than 25% within 4.5 h. This initial burst could be associated with the significant amount of drug just covering the outer surface of the nanofiber mats but not thoroughly encapsulated within, which can be easily solubilized in a favorable hydrophilic media like PBS. This initial burst may facilitate short-term localized and targeted formulations such as transdermal or topical (intra-oral as oral rinses). After 4.5 h, there was a slow and controlled drug release phase for up to 28 d of about 52% of pilocarpine. This secondary release phase may favor pilocarpine applications requiring a longer time in the mouth such as gel formulations to incorporate over stents or dentures.

Outcomes from the ex vivo study (Figure 3, Figure 4 and Figure 5) revealed that when exposed to fiber mats, the gland cellular compartments were all found at a proliferative/pro-mitotic state with negligible pro-apoptotic activity, indicating that the toxicity of our fiber mats is low and that there is no impairment to the biocompatibility of the SG organ and its cellular compartment. Hence, this had a positive biological impact on the epithelial growth of our SG ex vivo model. These low cytotoxicity findings are also corroborated by other reports using similar materials [25,31].

Current pilocarpine systemic formulations require multiple intakes per day (three times at least) leading to poor patient adherence to this drug formulation [6,8,12]. On the other hand, our localized pilocarpine-loaded nanofiber mats could potentially be used as a single-dose daily formulation containing the therapeutic amount of pilocarpine to enhance saliva secretion when administered locally via an intradermal, as well as through subcutaneous, transdermal or topical applications (e.g., oral rinses or gels for dentures). Our in vivo study is the first comparing localized and systemic pilocarpine formulations for xerostomia (Figure 6). Applying these pilocarpine-loaded nanofiber mats locally via an intradermal route over the in vivo gland could greatly increase the saliva flow rate earlier on (4.5 h) when compared to systemic pilocarpine only (Figure 6B). Furthermore, both in vivo and ex vivo studies confirmed the biocompatibility of the nanofiber mats and supported the gland homeostasis and secretion. These outcomes make this pilocarpine-loaded nanofiber mats promising for clinical use once a day since they can readily increase saliva flow well before 24 h (4.5 h collection time). The long-term effects of these pilocarpine mats are to be explored in different in vivo SG hypofunction models using a clinically relevant radiotherapy fractionated dose regimen. Despite this, acute single-dose radiotherapy SG models, like the one used herein, can better induce epithelial damage promptly within 48 h [32], and are pertinent to study localized short-term effects for our proposed once daily pilocarpine intradermal mat administration. Pre-clinical trials determining the pilocarpine release pharmacokinetics are the next step if intradermal applications are to be tested. If transdermal applications are to be investigated, then further studies are necessary with skin adhesive patches to assess pilocarpine penetration, partitioning, diffusion and permeation properties [14].

## 4. Materials and Methods

### 4.1. Fabrication of Electrospun Nanofiber Mats

PLGA (50:50) (Lactel absorbable polymers, Birmingham, MI, USA) with an inherent viscosity of 0.26–0.54 dL/g was dissolved in acetone (VWR Chemicals, Fontenay-sous-Bois cedex, France) at 30% (*w*/*v*) overnight. A pilocarpine 2% solution (Alcon, Vilvoorde, Belgium) and polyethylene glycol-400 (PEG-400, Sigma-Aldrich, St. Louis, MO, USA) were added to the PLGA solution at PLGA: pilocarpine: PEG-400 volume ratio of 1: 0.1: 0.002 and the mixture was then sonicated at 30% amplitude (Vibra-cell VCX 130, Sonics & Materials, Inc., Newtown, CT, USA) for a minute. For blank (unloaded) formulation, pilocarpine solution was replaced with distilled water. (Note: this final formulation was chosen after numerous rounds of optimization, using different solvents (dichloromethane (DCM), acetone, and ethanol) and mixing ratios/concentrations of each ingredients, to determine the most homogeneous and stable final polymeric solution/emulsion that does not phase separate. PEG known as a good surfactant was added to the solution to help increase the homogeneity and stability of emulsion.

After homogenization, solutions were loaded into a 5-mL syringe and the syringe was connected to the EHDA electrospinning setup to fabricate the fibers. The apparatus setup consisted of a stainless-steel needle/nozzle of inner diameter 0.72 mm (Popper and Sons, Lake Success, NY, USA), a syringe pump (KD Scientific, Holliston, MA, USA) to deliver polymer solution to the nozzle, a high voltage generator (Glassman High Voltage Inc., High Bridge, NJ, USA) to supply voltage to the nozzle via a crocodile clip, and a collecting stage at ground voltage. Upon steady state condition (i.e., the formation of Taylor cone at the tip of the nozzle), fiber sheets were collected in a glass Petri dish that was placed on the collecting stage. The dishes were subsequently wrapped with aluminum foil (since the drug is light-sensitive) and dried overnight in a vacuum oven to remove the residual solvent in the fibers. Next day, single-layer fiber sheets or mats were removed from the dishes and stored at 4 °C until use. Single-layer fiber mats were produced with different diameters (0.5, 1 and 2 mm) using a calibrated cylindrical puncher (Integra Miltex, Dutchess, NY, USA) as displayed in Table S1. This puncher allowed us to uniformly cut the mats to test the ex vivo biological effects with the different amounts/concentrations of biomaterials/pilocarpine, without changing their physical structure, fiber diameter, porosity and thickness. 

### 4.2. Physical and Chemical Characterization of Nanofiber Mats

#### 4.2.1. Scanning Electron Microscopy (SEM)

For SEM imaging, SEM metal stubs were placed directly under the Taylor-cone jet during electrospinning to collect representative fiber mat samples. The metal stubs were then sputter coated with Platinum (JFC-1300, JEOL, Tokyo, Japan) in vacuum at current intensity of 40 mA for 40 s. The coated samples were then viewed under the SEM (JEOL JSM 5600LV, Tokyo, Japan) to visualize the fibers morphology, distribution and pore size. To determine the diameter and porosity of the fibers, Image J software (NIH, Bethesda, MD, USA) was used. 

#### 4.2.2. Drug Loading Capacity and Encapsulation Efficiency

For this purpose, 10 mg of both unloaded and drug-loaded fiber mats were separately dissolved with 200 µL of DCM. Fibers were then vortexed for 30 s until full dissolution. Next, 5 mL of distilled water was added to the dissolved fibers, vortexed for 30 s, and centrifuged at 10,000 rpm for 10 min. Afterwards, the fibers were left for 1.5 h to allow for the separation of the organic and aqueous phases. Then, 3 mL of samples were collected from the aqueous phase. The samples were then analyzed for pilocarpine concentration by UV-Vis spectrophotometry (UV 1800, Shimadzu, Japan) at 215 nm (greatest sensitivity and maximum absorbance values; results not shown), with a standard curve having a good linear fitting (*R^2^* = 0.9965) within the pilocarpine concentration range of 5–40 μg/mL [33]. Unloaded fibers were used as the blank/reference in this analysis. These experiments were conducted in triplicate. Drug loading capacity was calculated using the following formula: (drug weight/(drug weight + PLGA weight + PEG-400 weight)) × 100%. The encapsulation efficiency (*EE*) was quantified using the following formula as per previous report (Kao, Lin et al. 2006): *EE* = [(Total pilocarpine-Free pilocarpine amount)/Total pilocarpine amount)] × 100%. 

#### 4.2.3. Degradation Studies for the Pilocarpine Solution

The pilocarpine solution was tested for degradation in diluents such as 1× phosphate buffered saline (PBS) and ultra-pure water (MilliQ water, Sigma-Aldrich, Saint Louis, MO, USA) as this is essential to validate the drug release experiments in vitro [33,34]. For this purpose, pilocarpine solution was dissolved in 5 mL MilliQ water and 1x PBS to have a final concentration of 40 µg/mL. Solutions were vortexed for 30 s and placed in the rotary incubator at 37 °C, 100 rpm. At pre-determined time intervals (4.5 h, 1 d, 2 d), 3 mL of the drug solutions (in the different diluents) were drawn and analyzed within a wavelength ranging from 200 nm to 300 nm in the UV-Vis spectrophotometer. Reference samples of pure MilliQ water and 1× PBS only were used to blank the pilocarpine containing solutions.

### 4.3. Pilocarpine Drug Release Analysis from Loaded Nanofiber Mats

Fabricated nanofiber mats (pilocarpine-loaded and unloaded) weighing 20 mg each were used to analyze the drug release in vitro in the short and long term. Then, 20 mg fibers were placed in 4 mL of 1× PBS (pH 7.4) in 15-mL Falcon tubes. Tubes were vortexed for 30 s and placed in an orbital shaker bath (GFLVR 1092, Burgwedel, Germany) at 37 °C, 100 rpm. At pre-determined time intervals (4.5 h, and 1, 2, 3, 5, 6, 9, 12, 15, 19, 23, and 28 d), the tubes were subjected to centrifugation at 10,000 rpm for 10 min. After centrifugation, the supernatant (3.3 mL) was carefully collected from each tube into a new tube and stored at −20 °C for further pilocarpine quantification. Fresh 1× PBS (3.3 mL) was then added to the original sample tubes, vortexed for 30 s and placed back in the incubator. This procedure was repeated for all collection time points. Three biological replicates were run for both unloaded and loaded nanofibers for each time point. The pilocarpine concentration in the collected samples was again measured by UV-Vis spectrophotometer as described above. For each collection time point, the average absorbance readings of the unloaded nanofiber were subtracted from each of the loaded nanofibers, and then these were added up to calculate the cumulative drug release since the first collection time at 4.5 h.

### 4.4. Ex Vivo SG Organ Culture Studies

#### 4.4.1. SG Organ Culture Model

Pregnant ICR mice were used as per the approved National University of Singapore IACUC protocol no. 2014-00306. Mice at embryonic day 15, after epithelial differentiation occurs to become an adult organ, were selected. Major salivary glands including submandibular and sublingual glands were isolated from ICR mouse embryos using a microdissection technique under a stereozoom microscope (M80, Leica, Wetzlar, Germany) as per previous reports [35,36]. The glands were then placed onto polycarbonate filter papers (Whatman™ Nuclepore™ Track-Etched Polycarbonate Membrane Filter, Sigma-Aldrich, Saint Louis, MO, USA). A growth medium was added (200 µL per culture plate), which was composed of DMEM/F12 media, penicillin‒streptomycin (1% *v*/*v*), vitamin C (0.2% *v*/*v*), and transferrin (0.02% *v*/*v*) (Thermo Fisher Scientific, Waltham, MA, USA). Four whole salivary glands were cultured per treatment group and incubated at 37 °C with 5% CO_2_.

#### 4.4.2. Cytotoxicity of Pilocarpine

The gland growth media of the ex vivo organ culture was supplemented with clinically relevant concentrations of pilocarpine ranging from 0.1 to 25 µg/mL. The cultures were then incubated as previously. Epithelial cellular growth was analyzed at baseline (t_0_), day 1 and day 3 by taking bright field microscopy images at 3.2× magnification with the above stereozoom microscope set up. The total number of epithelial buds per gland was quantified from those images using Image J (NIH). The growth index for each gland was determined by normalizing the number of epithelial buds at day 1 and day 3 to the ones at baseline [36]. The growth index was used as a readout for organ cytoxicity. The positive control glands were those grown with the growth media only (without pilocarpine supplementation). The negative control glands were glands subjected to cytotoxic damage by gamma rays (7 Gy dosage) at baseline. 

#### 4.4.3. Biocompatibility of Nanofiber Mats

Punched nanofiber mats measuring 0.5, 1, and 2 mm were sterilize d under UV light for at least 6 h before utilization. To test the biocompatibility, mats were added to the growth media in the ex vivo SG organ cultures. These cultures were incubated for 5 d, and at each day, the viability index that measures epithelial cellular growth was determined for each gland as described above. Same positive and negative controls glands were used as before. Unloaded discs were used to make sure the unloaded nanofibers by itself are not causing any organ cytotoxicity. Three independent biological experiments were performed and four whole glands were used for each experiment. 

#### 4.4.4. Salivary Gland Cellular Analysis for Proliferation and Apoptotic Activity

Whole-mount immunofluorescence staining was used to evaluate and quantify the proliferative and apoptotic cell subpopulations in the ex vivo SG organ culture according to previous protocols [25,26]. Briefly, whole SG treated for 5 d with loaded nanofibers discs (0.5, 1, 2 mm in diameter) from the previous experiments were fixed in 4% paraformaldehyde followed by 1× PBS washing steps. Then, fixed glands were permeabilized, and blocked using 10% donkey serum and 5% bovine serum albumin for 2 h. This was followed by overnight incubation at 4 °C with primary antibodies anti-Ki67 (1:200 dilution, Cat no. 556003, BD Pharmingen, San Jose, CA, USA) and anti-cleaved Caspase 3 (1:200 dilution, Cat no. 9664S, Cell Signalling, Danvers, MA, USA). After washing, incubation with respective secondary antibodies followed (1:200 dilution, Alexa Fluor 488 and Alexa Fluor 635, Thermo Fisher Scientific). Rhodamine-labelled peanut agglutinin (1:200 dilution, Vector Lab, Burlingame, CA, USA) was used to stain the acinar and ductal branched epithelial structures of each gland. A nuclear fluorescent dye (Hoechst 33342, Invitrogen, Carlsbad, CA, USA) was used to counterstain the nuclei. Whole glands were then mounted on glass slides in resin mounting media using spacers to avoid gland crushing by glass slide. Slides were visualized, scanned and analyzed with Leica DMI-8 fluorescence microscope with a z-motorized axis and Leica LAS-X software (Leica Microsystems, Wetzlar, Germany) to obtain a z-stack of images for each gland with 20 µm z-steps. Maximum intensity projections were further produced at 10× and 20× magnifications. Automated cell counts for each gland z-stack were done using Image J (NIH, USA) for immuno-fluorescently labeled Ki-67+ and Caspase 3+ cells, and for all cells stained with the nuclear dye. Ki-67+ and Caspase 3+ cell counts for each gland were normalized to the total number of cells (stained with the nuclear dye).

### 4.5. In Vivo SG Hypofunctional Model

To generate hypofunctional SG with xerostomia measurable signs, a single acute dose of gamma radiation (30 Gy) was given to eight-week-old C57BL/6 mice as per a published protocol [37]. This protocol was approved by the National University of Singapore IACUC application no. 2014-00306. All in vivo experiments complied with the ARRIVE guidelines and were carried out in accordance with the National Institutes of Health guide for the care and use of laboratory animals (NIH Publications No. 8023, revised 1978). Animals were housed in a climate- and light-controlled environment and allowed free access to food and water at the bottom of the cage. One day after radiation was delivered, our localized pilocarpine nanofiber formulation was administered over the submandibular glands (via an intradermal route) and compared with systemic pilocarpine (via an intraperitoneal route, Sigma). The oral pilocarpine clinical formulation (available in the market) could not be given according to the IACUC committee, due to the unpredictability and life-threatening adverse side effects in rodents. The following experimental mouse groups were tested on a daily basis as per the acute SG dry mouth model: (A) Non-irradiated control (nonIR, *n* = 3); (B) Irradiated control (IR, *n* = 5); (C) Irradiated and treated daily with 0.5 mm diameter pilocarpine-loaded nanofibers (PNM, *n* = 5), the ones that performed effectively in the ex vivo biocompatibility tests; (D) Irradiated and treated daily with systemic pilocarpine as a positive/conventional control treatment (SP, *n* = 5). Intraperitoneal ketamine (10 mg/mL) and xylazine (1 mg/mL) were administered before the treatment was delivered to minimize animal discomfort. As for the nanofiber composition and formulation, the sterilized ones used in the abovementioned ex vivo studies were administered here: first these were placed in a syringe vessel with a 21G needle, and a sterile PBS 1× solution was added as a vehicle.

Whole stimulated saliva was collected at 4.5 h and 24 h after nanofiber administration. Saliva was extracted by capillarity every 3 min for 15 min using a 75-mm hematocrit tube (Drummond, Scientific Company, Broomall, PA, USA) [38]. These tubes were then placed in 1.5-mL pre-weight Eppendorf tubes, which were then weighed to determine the saliva secretion rate. To determine the percentage of secreted saliva relative to the control (NonIR), the saliva secretion rate was normalized to the average of saliva secreted by the NonIR group. Submandibular glands were surgically removed and freshly dissected in a stereozoom microscope and their weight was measured before fixation. Glands were then fixed with paraformaldehyde 4% overnight at 4 °C in a rotating shaker, followed by several washing steps with PBS and incubation with 30% cold sucrose (*w*/*v*) solution in 0.01 M PBS for 3–4 d. Then, after paraffin embedding, 4-μm gland sections were made and stained with hematoxylin and eosin and visualized with bright-field microscopy at 20–40× magnification.

### 4.6. Statistical Analysis

Data are presented as mean ± standard deviation (SD) from 3–12 independent samples, except for the in vivo experiments, where the standard error of the mean (SEM) was used. All statistical analyses were conducted using Prism version 6 (GraphPad Software, Inc., San Diego, CA, USA). All datasets were tested for normality. Student *t*-tests were performed for two-group comparison: unpaired with Welch correction for in vivo study outcomes, or paired for time-dependent experiments for drug release profile experiments. A one-way ANOVA with Dunnet post hoc tests for comparison of three or more experimental groups with a positive control for the case of ex vivo studies. The significance level for all experiments was set at *p* < 0.05. 

## 5. Conclusions

In this study, the ultimate aim was to determine whether an intradermal pilocarpine-loaded PLGA/PEG nanofiber mat can be utilized to release pilocarpine while supporting the SG organ viability and stimulating saliva secretion.

The intradermal pilocarpine-loaded nanofiber mats ranging from 0.5 to 1 mm diameter induced the highest SG growth and cell proliferation with negligible cytotoxicity (apoptosis) in ex vivo SG models. In the acute dry mouth in vivo model, the daily intradermal application of 0.5 mm diameter pilocarpine-loaded nanofiber mats stimulated a higher saliva secretion before 24 h as compared to the conventional systemic pilocarpine. After 24 h, saliva secretion was comparable to systemic pilocarpine.

Thus, this pilocarpine delivery system is promising for potential clinical use as an intradermal formulation for the early and prompt treatment of xerostomia before and after 24 h of application. These formulations will be particularly useful in patients who have poor saliva secretion with conventional saliva stimulants.

## Supplementary Materials

The following are available online at http://www.mdpi.com/1422-0067/20/3/541/s1.
