# Supplementary material for: Localized Delivery of Pilocarpine to Hypofunctional Salivary Glands through Electrospun Nanofiber Mats: An Ex Vivo and In Vivo Study
Source: Int J Mol Sci. 2019 Jan 28;20(3):541. doi: 10.3390/ijms20030541 (PMC6387464; doi:10.3390/ijms20030541)
Supplement: Supplementary file 1 [file ijms-20-00541-s001.pdf]

Supplementary Table S1. Table displaying the three fabricated nanofiber mats with different diameters (0.5, 1 and 2mm) and similar internal morphology and structural properties (thickness, mesh porosity, and nanofiber diameter).

**Nanofiber mat diameters** (mm)	**Total pilocarpine** (µg)	**Total nanofiber** (µg)
0.5	0.21	25
1	0.42	50
2	0.84	100

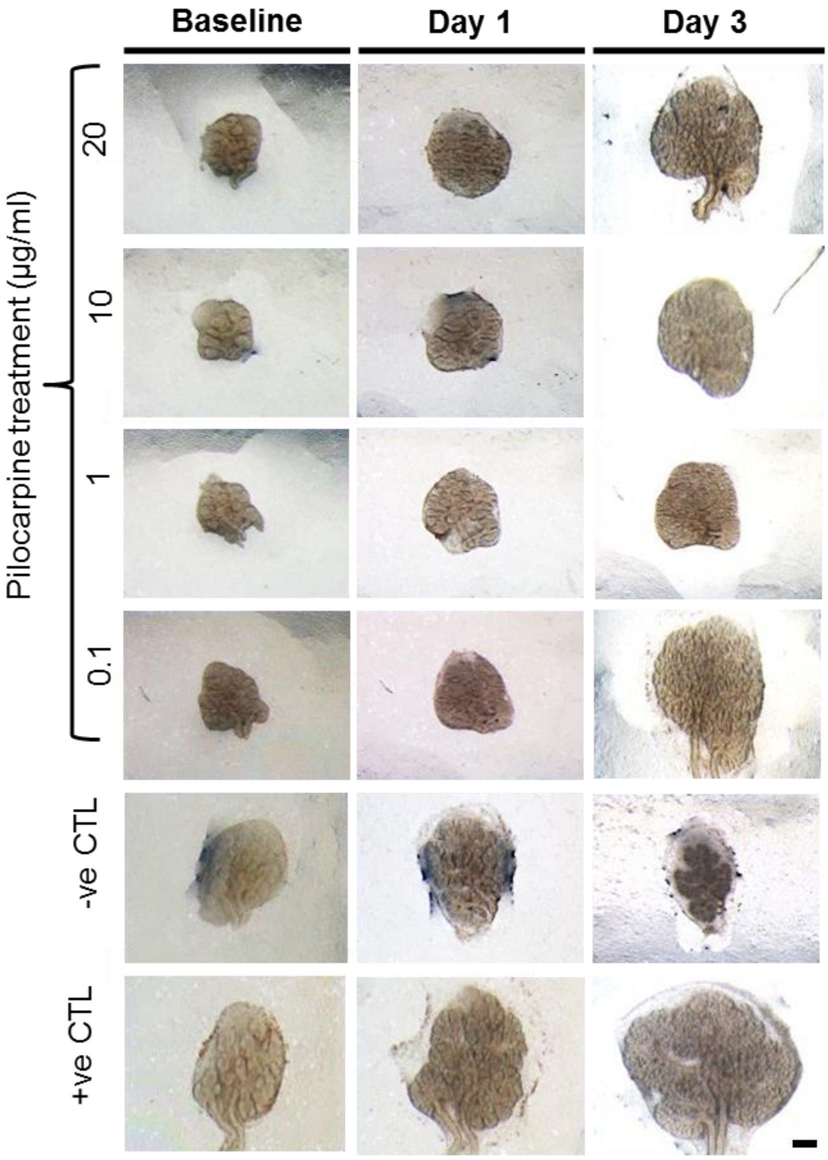

Supplementary Figure S1. Salivary gland growth shown in brightfield microscopy before and after treatment with pilocarpine-loaded nanofiber discs in the *ex vivo* SG culture model. Legend: +ve CTL: treated with growth media only. -ve CTL: treated with gamma radiation in growth media only to induce cytotoxicity. Mag.: 3.2x. Scale bar: 100µm
